# Rapid Diagnosis of Nonconvulsive Status Epilepticus Using Reduced-Lead Electroencephalography

**DOI:** 10.5811/westjem.2015.3.24137

**Published:** 2015-04-06

**Authors:** Jay M. Brenner, Paul Kent, Susan M. Wojcik, William Grant

**Affiliations:** State University of New York Upstate Medical University, Departments of Emergency Medicine and Neurology, Syracuse, New York

## Abstract

**Introduction:**

Electroencephalography (EEG) is indicated for diagnosing nonconvulsive status epilepticus (NCSE) in a patient who has altered level of consciousness after a motor seizure. A study in a neonatal population found 94% sensitivity and 78% specificity for detection of seizure using a single-lead device. This study aims to show that a reduced montage EEG would detect 90% of seizures detected on standard EEG.

**Methods:**

A portable Brainmaster EEG device was available in the emergency department (ED) at all times. Patients presenting to the ED with altered mental status and known history of seizure or a witnessed seizure having a standard EEG were eligible for this study. The emergency physician obtained informed consent from the legally authorized representative (LAR), while an ED technician attached the electrodes to the patient, and a research associate attached the electrodes to the wiring routing to the portable EEG module. A board-certified epileptologist interpreted the tracings via the Internet. Simultaneously, the emergency physician ordered a standard 23-lead EEG, which would be interpreted by the neurologist on call to read EEGs. We compared the epileptologist’s interpretation of the reduced montage EEG to the results of the 23-lead EEG, which was considered the gold standard for detecting seizures.

**Results:**

Twelve of 12 patients or 100% had the same findings on reduced-montage EEG as standard EEG. One of 12 patients or 8% had nonconvulsive seizure activity.

**Conclusion:**

The results are consistent with prior studies which have shown that 8–48% of patients who have had a motor seizure continue to have nonconvulsive seizure activity on EEG. This study suggests that a bedside reduced-montage EEG can be used to make the diagnosis of NCSE in the ED. Further study will be conducted to see if this technology can be applied to the inpatient neurological intensive care unit setting.

## INTRODUCTION

Patients having a seizure compose one million of all emergency department (ED) visits in the U.S.^1^ Approximately 6% of these seizures are prolonged or recurrent without a return to baseline and are designated as status epilepticus (SE), as defined by 30 minutes of continuous seizure activity or a series of seizures without return to full consciousness between the seizures.^2^–^4^ Further, 8–48% of these patients with SE will have nonconvulsive status epilepticus (NCSE) diagnosed by electroencephalography (EEG).^5^,^6^ The mortality of NCSE can exceed 30% if the seizure lasts more than one hour.^7^

Approximately 2% of EDs in the U.S. have EEG technicians available to obtain tracings and neurophysiologists to interpret EEG 24 hours a day seven days a week, and studies have shown that it takes three hours on average to obtain and interpret an EEG in the ED.^8^,^9^ Because permanent brain damage may occur after only 30 minutes in NCSE it would be ideal to have a quicker means of determining if patients are in NCSE at ED presentation. Earlier recognition of NCSE may save lives and costs by diagnosing a previously unrecognized cause of a patient’s altered mental status (AMS) and/or by avoiding overtreatment of presumed seizures.

Standard EEG in the U.S. requires active electrodes and a specially-trained EEG technician to obtain tracings. Therefore, there have been several attempts to conduct bedside EEGs with passive electrodes to decrease the time interval of arrival to interpretation by a general healthcare technician in the ED. One study researched the use of a helmet with EEG electrodes, but this approach was cumbersome.^10^ BrainScopes, a company dedicated to EEG applied research, sponsored a study evaluating a device that supplied a red light/green light function to indicate based on a quantified electronic algorithm if a patient was exhibiting seizure activity. The removal of the human interpreter led to results doubted by neurophysiologists. While relying on algorithmic interpretations may be helpful in situations with untrained care takers, in the ED there are trained personnel able to interpret more complex diagnostic outputs that could prevent missed diagnoses and inappropriate overcalls. Therefore, there is still a need for the development of rapid bedside testing of patients with AMS for possible NCSE.

The objective of the study was to determine if it is possible to use a passive electrode reduced-lead EEG in the ED to determine if patients with AMS are experiencing nonconvulsive status epilepticus at ED presentation. The proposed model employed six leads, two frontal, two temporal, a ground, and a reference, designed to capture the areas where 80% of seizures originate.

## METHODS

**Figure 1:**
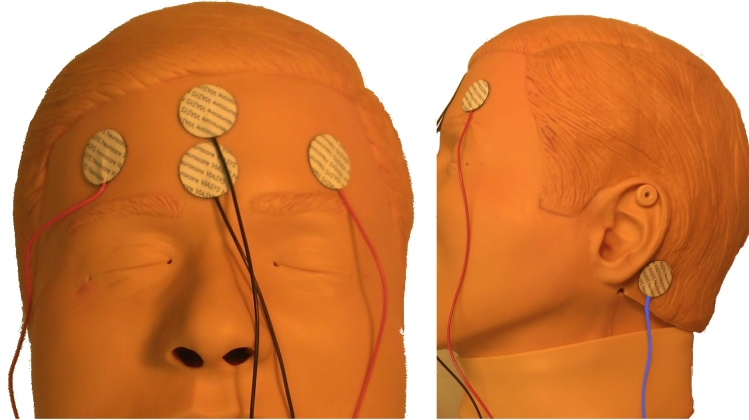
Electrode placement.

This study was a convenience sample of adult patients presenting to the ED with AMS. These patients were screened for eligibility in the study when research associates or the study PI were available. We included patients with a history of seizures or witnessed seizures, having a standard EEG ordered as part of their care in the ED for evaluation of persistent altered level of consciousness (ALOC) and who had a family member available to give pre-consent. Patients under the age of 18 were excluded, as well as patients who had no known history of seizures or witnessed seizures or persistent ALOC. A portable Brainmaster EEG device was available in the ED at all times for recording of the reduced-lead EEG. Immediately following pre-consent the research associate prepared the Brainmaster EEG and notified the ED technician to apply the electrodes. The electrode placement is shown in Figure 1. The Brainmaster EEG system provided the capabilities for a neurophysiologist to gain real-time remote Internet access to view and interpret the reduced-montage EEG tracings. Time was recorded from the completion of study consent to start of the Brainmaster EEG recording and then interpretation of the study neurophysiologist. Simultaneously, the emergency physician ordered a standard 23-lead EEG, which would be interpreted by the neurologist on call. We compared the neurophysiologist’s interpretation of the reduced-montage EEG to the results of the 23-lead EEG, which was considered the gold standard for detecting seizures. Patients were post-consented following obtaining baseline mental status. We excluded any patients not willing to consent to the study at that time, and their data was not used. Following their ED visits, we reviewed patients’medical records to determine the results of the clinical 23-lead EEG. This study was reviewed and approved by the SUNY-Upstate Medical University Institutional Review Board.

## RESULTS

**Figure 2:**
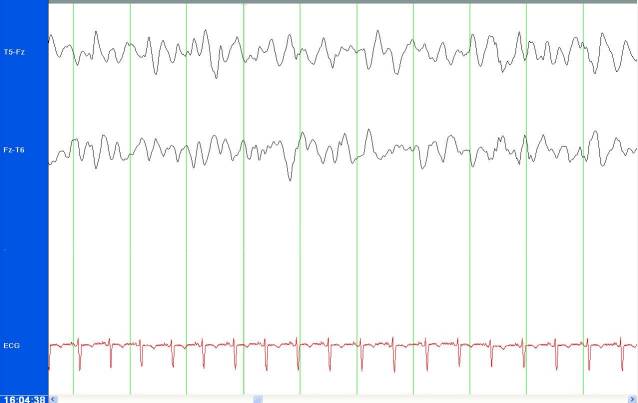
Tracings from Brainmaster reduced-lead electroencephalogram showing periodic lateralized epileptiform discharges.

We enrolled 12 patients from February 10, 2010–July 19, 2011. The study patients were 50% male with a median age of 51.5 years (range 25–81 years). The time from study consent (surrogate for ordering an EEG) to beginning of the study EEG recording was a median of 10 minutes (range 5–40 min) (n=11) and median of 38 min (range 10–135 min) (n=8) until neurophysiologist interpretation. For all 12 patients, or 100% of the time, the research neurophysiologist’s interpretation of the reduced-lead EEG and the clinical neurophysiologist interpretation of the standard EEG were the same for whether or not the patient was in NCSE. The demographics for the 12 included patients are shown in the Table. Only one of 12 patients or 8% was determined to have nonconvulsive seizure activity. The resulting tracings from the Brainmaster reduced-lead EEG for a patient determined to be in NCSE is shown in Figure 2.

## DISCUSSION

The current approach to diagnosing NCSE in the ED setting is to obtain a standard EEG, which has been shown to take three hours on average nationwide. As permanent brain damage in incompletely or inadequately diagnosed patients may occur after 30 minutes of uncontrolled seizures, it is imperative to develop modalities to bridge this gap. Bleck states that EEG is crucial in the diagnosis and classification of potential seizures in his review of continuous EEG monitoring in the ICU.^11^ It is time for this technology to come to the ED.

The best justification for the use of reduced-lead EEG is found in a study of the neonatal population. While newborns have very different EEG tracings, they have fairly similar obstacles to obtaining and interpreting standard EEG as adult patients in an ED. Shellhaas and Clancy detected 94% of seizures with a single-lead EEG compared to the standard 10-lead neonatal EEG.^12^ This study established the baseline expectation for our study. While this study represents only a small sample, our results were consistent with prior studies, which have shown that 8–48% of patients who have had a motor seizure continue to have nonconvulsive seizure activity on EEG.

Ultimately, emergency physicians could perhaps interpret the screening bedside reduced-lead EEG themselves to make clinical decisions in real time. We conducted a study on emergency medicine residents in our simulation lab to assess their comfort level with interpreting EEG in the case of a patient with NCSE. They averaged about a 2 on a scale of 1 to 5, with 5 being very comfortable with a past experience average of about one day during medical school. If we were to expand training, as we have with electrocardiogram interpretation, comfort levels and reliability would theoretically improve.

In the meantime, however, an epileptologist (a neurologist with fellowship training in epilepsy) is the most appropriate physician to interpret EEG tracings from either a standard 23-lead EEG or our reduced-lead EEG device. Several studies have looked into the use of standard EEG in the ED for evaluation of seizure, but none discuss the use of reduced-lead EEG. Three studies investigated the use of reduced-lead EEG in the ICU. Two of them reported on the sensitivity and specificity as compared to standard EEG. One found 68% sensitivity and 98% specificity for seizure detection using a four-channel device.^13^ Another found 54% sensitivity and 100% specificity.^14^ Another study showed that neurophysiologists had 70% sensitivity and 96% specificity for seizure detection when interpreting archived EEGs presented to them with reduced-lead montages.^15^

Although our study did not explicitly study the cost effectiveness of reduced lead EEG, we ought to acknowledge that the reduced-lead EEG device used in our study cost $2,500. The standard EEG device cost approximately 20 times that amount.

Our pilot study suggests that reduced-lead EEG may be quicker than standard EEG and may be sufficiently sensitive and specific to diagnose NCSE. While not intended to be as comprehensive as standard EEG, reduced-lead EEG may be useful as a screening tool in the acute care setting such as the ED. Even in a resource-poor facility, Internet access to these tracings may open the potential for neurology telemedicine coverage to improve patient care.

## LIMITATIONS

This study is limited by the size of the sample of patients. It had been intended to recruit 120 subjects to reach statistical significance of feasibility based on the prior neonatal study. It was difficult to enroll patients because standard EEG is performed infrequently in the ED. When EEG was ordered, it was often by the neurologists, who did not necessarily communicate this with the emergency physicians or research associates.

Real-time Internet access to the tracings was not always obtained, therefore not providing an assessable time frame for off-site neurophysiologist interpretation in some cases. Also, the variation from an n of 12 to an n of 8 was due to research associates’ failure to capture time data. Standard EEG time date was not captured at the time of the study, and unfortunately because of a vendor change for the standard EEG data, this is not available retrospectively either.

The study equipment used required access to a wired Internet port and IP addresses, which proved more difficult to find throughout the ED than anticipated. For future studies if immediate Internet access and interpretation were necessary, a wireless Internet connection would be necessary.

## CONCLUSION

This study suggests that the use of a bedside reduced-montage EEG as a screening tool may be feasible in the ED to make the diagnosis of nonconvulsive status epilepticus in patients with AMS on arrival.

## Figures and Tables

**Table:** Demographics, medications, and comorbidities of study patients.

Age	Sex	Current medications	Comorbidities
80	F	Levetiracetam, levothyroxine, lisinopril, alendronate, aspirin, celecoxib, ranitidine	Seizure disorder, hypothyroidism, hypertension, transient ischemic attack, gastroesophageal reflux disease, migraine
44	F	Cyclobenzaprine	None
48	M	Aspirin, hydrocodone, duloxetine, propranolol, escitalopram, clonazepam, oxycodone, gabapentin	Bipolar disorder, depression, transient ischemic attack, posttraumatic stress disorder, nephrolithiasis
54	M	None	Anxiety, depression, opioid addiction, hypertension, alcohol dependence
49	M	Clonazepam, duloxetine, fentanyl, hydrochlorothiazide, lamotrigine	Hyperammonemia, depression, anxiety, bipolar disorder
62*	F	Levetiracetam, oxcarbazepine, phenytoin, glargine, atenolol, albuterol, Ipatropium bromide and albuterol, venlafaxine, pantoprazole	Encephalitis, chronic obstructive pulmonary disease, lymphoma, hypertension, type 2 diabetes mellitus
55	M	Allopurinol, aspirin, divalproex sodium, lisinopril, metoprolol, risperidone	None
81	F	Not documented	Not documented
33	M	Docusate, phenytoin, simvastatin, hydroxyzine, ezetimide, sertraline, olanzapine	Stroke
26	M	Carbamazepine, methotrexate	None
60	F	Ciprofloxacin, rasuvostatin, l-methylfolate, sumatriptan, clonazepam, levothyroxine	Depression, asthma, Hashimoto’s thyroiditis, migraines, restless leg syndrome, hydronephrosis
25	F	None	None

* Patient with nonconvulsive status epilepticus.
